# Mode-evolution-based polarization rotation and coupling between silicon and hybrid plasmonic waveguides

**DOI:** 10.1038/srep18378

**Published:** 2015-12-18

**Authors:** Sangsik Kim, Minghao Qi

**Affiliations:** 1 School of Electrical and Computer Engineering and Birck Nanotechnology Center, Purdue University, West Lafayette, IN 47907 USA

## Abstract

Hybrid plasmonic (HP) modes allow strong optical field confinement and simultaneously low propagation loss, offering a potentially compact and efficient platform for on-chip photonic applications. However, their implementation is hampered by the low coupling efficiency between dielectric guided modes and HP modes, caused by mode mismatch and polarization difference. In this work, we present a mode-evolution-based polarization rotation and coupling structure that adiabatically rotates the TE mode in a silicon waveguide and couples it to the HP mode in a strip silicon-dielectric-metal waveguide. Simulation shows that high coupling factors of 92%, 78%, 75%, and 73% are achievable using Ag, Au, Al, and Cu as the metal cap, respectively, at a conversion length of about 5 *μ*m. For an extremely broad wavelength range of 1300–1800 nm, the coupling factor is >64% with a Ag metal cap, and the total back-reflection power, including all the mode reflections and backscattering, is below *−*40 dB, due to the adiabatic mode transition. Our device does not require high-resolution lithography and is tolerant to fabrication variations and imperfections. These attributes together make our device suitable for optical transport systems spanning all telecommunication bands.

Silicon (Si) photonics, which utilizes core and cladding materials that are compatible with complementary metal-oxide semiconductor (CMOS) technology for light guiding, modulation and detection, is promising for high-bandwidth communication and interconnection between computing nodes, due to the well-developed, low-cost CMOS manufacturing^1^,^2^,^3^,^4^,^5^. Further scaling down Si photonic devices for on-chip interconnects or information processing is hampered by, among others, the diffraction limit. Plasmonics in metallic nanostructures can confine light at subwavelength scale with enhanced field intensity^6^,^7^,^8^,^9^,^10^, thus in many cases breaking the diffraction limit and increasing the operating bandwidth^11^,^12^,^13^,^14^. The required power for an electro-optical modulation can also be reduced with increased light-matter interaction and reduced parasitic capacitance^15^. However, the use of plasmonic nanostructures is generally limited by the high optical loss in metals^16^,^17^.

Recently, hybrid plasmonic (HP) waveguide structures have been proposed to reduce the high propagation loss of plasmonic waveguides^8^,^9^. A typical HP waveguide consists of a Si core and a metal cap, in between which is a gap filled with a low-index material such as SiO_2_. When the gap is small, e.g. 50 nm, about 60% of the electromagnetic field can be confined within the low-index material, resulting in both a moderate propagation loss and a high field confinement in a low-index medium^8^,^18^. Electro-optical modulators^15^, nano-lasers^10^, ring-resonators^19^, and nonlinear optical devices^20^, based on, or related to HP waveguides have been demonstrated. Recently, various types of HP waveguide configurations based on nano-ridges^21^, metal ribbon buried slots^22^, and Si-on-nitride gap plasmon^23^ have been proposed at mid-infrared (MIR) to take advantage of the reduced propagation loss in plasmonic devices at these longer wavelengths, thus making HP waveguides attractive for MIR applications such as chemical and biological sensing^24^,^25^,^26^. To capitalize on these promising attributes of HP waveguides and integrate this platform into practical systems, meanwhile, requires a highly efficient and robust coupler between HP and Si waveguides to interface with Si photonics.

Previously, several schemes of couplers from Si waveguide to dielectric-loaded plasmonic waveguide^27^, metal-insulator-silicon-metal waveguide^28^, and HP waveguide^29^ have been proposed or demonstrated. Among them, the HP waveguide coupler design shows a broadband performance with a maximum coupling efficiency of about 70% at a gap size of 50 nm, from TM mode (electric field perpendicular to the surface) to HP mode^29^. However, in Si photonic devices, TE mode (electric field parallel to the surface) is dominant due to its higher confinement and ease of fabrication. Thus, to fully utilize the HP waveguide in Si photonic devices, it is essential to have an efficient coupler between TE mode and HP mode, that is, to rotate the TE mode in a Si waveguide and simultaneously couple the rotated mode to the HP mode. Achieving simultaneously high coupling efficiency (>90% with metal propagation loss), low reflection (<30 dB as on-chip isolators are not readily available), broad bandwidth (preferably > 500 nm to cover all telecommunication bands), and high tolerance to fabrication imperfections is highly desirable for such devices to be incorporated into large-scale silicon photonics integration and manufacturing. Unfortunately, to the best of our knowledge, no such couplers have been reported.

In purely dielectric photonic systems, mode-evolution based polarization splitter and rotators have been demonstrated with high performance^30^,^31^,^32^. Such devices usually include tapers to adiabatically splitting or rotating modes. However, simply tapering out the metal layer into sharp tips in a similar fashion does not achieve mode-evolution. Rather, the tapered sharp metal tip focuses and confines the light into the plasmonic mode^33^,^34^.

Several polarization rotators consisting of plasmonic structures have been proposed^13^,^35^ or fabricated^14^ to reduce the device length for Si photonic applications. Such devices utilize asymmetric HP structures, covering the top and one side-wall of Si waveguide with oxide and metal^35^ or tapering out the metal layer of HP waveguide^13^,^14^. However, these designs did not focus on the coupling to hybrid modes and are based on mode-coupling^35^ or a combination of mode-coupling and mode-evolution^13^,^14^. For example, in Fig. 2 of ref. 13, the oscillation in polarization extinction ratio (PER) from 2 *μ*m to 7 *μ*m for the case of 90 nm spacer thickness is a signature of mode-coupling. Therefore it remains interesting to find a scheme to achieve pure mode-evolution when plasmonic effect is involved, and conduct a systematic investigation on the mechanism of transition between a dielectric mode and a HP mode.

In this paper, we present a truly mode-evolution-based polarization rotation and coupling (PRC) scheme that rotates the TE mode adiabatically and couples the rotated mode to the HP mode. When material absorption by metals is ignored, the coupler achieves almost 100% of efficiency with a proposed perfectly-phase-matched taper design. The performance of different metal caps (Cu, Au, Ag, and Al) with a linear Si taper is evaluated, and their spectral responses are assessed. Compared to mode-evolution structures in purely dielectric systems, our coupler has an upper limit of 92% efficiency when using silver as the metal cap, but it does have a much more compact size (~5 *μ*m) and broader bandwidths (~500 nm), which are desirable in future dense optical integration.

## Device design

**Figure 1:**
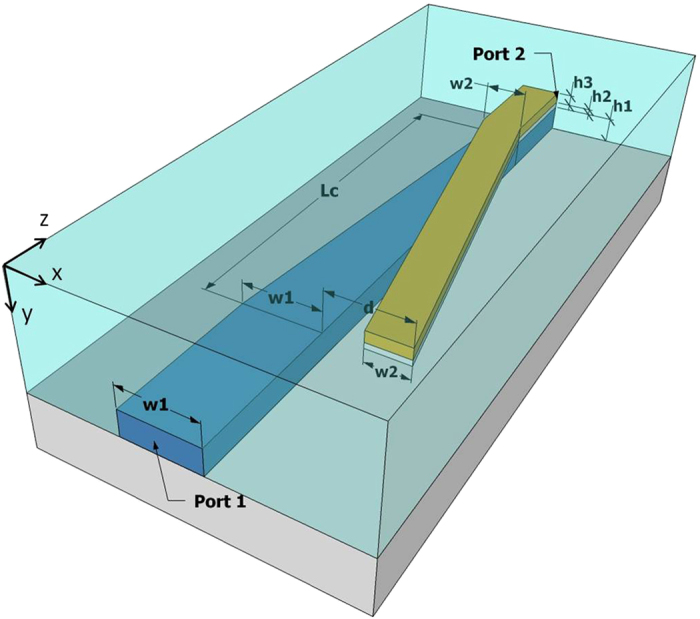
Schematic and geometric parameters of the mode-evolution-based polarization rotation and coupling structure. Metal cap and Si core are colored in yellow and blue, respectively, and SiO_2_ substrate and cladding are colored in grey and cyan, respectively.

Figure 1 shows the schematic view of the mode-evolution-based PRC structure, and geometric parameters; *w*_1_ and *w*_2_ are the widths of Si waveguide at port 1 and HP waveguide at port 2, respectively, and *h*_1_, *h*_2_, and *h*_3_ are the heights of Si core, SiO_2_ spacer, and metal cap, respectively. The conversion/coupling region has a length of *L*_*c*_, and starts when the lateral shift between the metal cap and Si core is *d*, and ends when the metal cap is exactly on top of the Si core. Through the conversion length *L*_*c*_, the width of the Si core waveguide tapers from *w*_1_ to *w*_2_, while that of the metal cap is fixed to *w*_2_.

**Figure 2:**
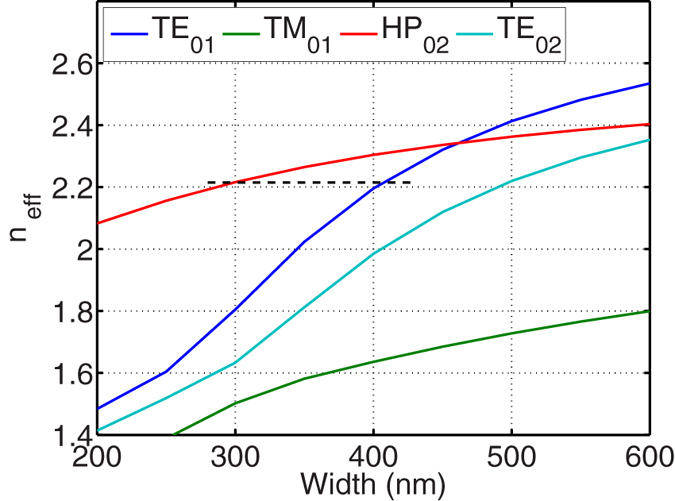
Effective refractive index of each mode, as a function of Si waveguide width, at *i*-th port (subscript); TE and TM modes at port 1 are plotted with blue and green lines respectively, and HP and TE modes at port 2 are plotted with red and cyan lines, respectively.

We first calculate and plot the effective refractive indices of each mode in Fig. 2 as a function of Si core width (*w*_1_ or *w*_2_). Finite element method (FEM) is used for the calculation with a free space wavelength of *λ*_0_ = 1550 nm. The refractive indices of Si and SiO_2_ are chosen to be *n*_Si_ = 3.445 and 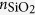 = 1.445. For the metal cap we choose Cu for its CMOS compatibility, and the dispersive complex refractive index of Cu is used^36^. Waveguide heights are fixed to *h*_1_ = 220 nm, *h*_2_ = 30 nm, and *h*_3_ = 100 nm. Here, the thickness of SiO_2_ spacer is chosen to strike a balance between the field confinement and propagation length for the subsequent hybrid mode^8^,^9^,^18^. In general, a small spacer thickness is desirable as it would lead to strong field confinement in the spacer, which is one of the motivations to incorporate hybrid modes into the silicon photonics platform^8^,^9^,^18^. A thinner spacer also requires an optimized coupler design as direct butt coupling would cause stronger reflection and scattering. Other thickness can also be selected to promote confinement over propagation loss or vice versa, and the coupler can be designed accordingly. The thickness of Si core is chosen to be compatible with common silicon-on-insulator (SOI) wafers. TE_01_ (blue) and TM_01_ (green) are the fundamental TE and TM modes at port 1, respectively, and HP_02_ (red) and TE_02_ (cyan) are the fundamental HP and TE modes at port 2, respectively. Our goal is to match the propagation constants (phases) between TE_01_ and HP_02_, while having a wide phase gap between TE_01_ and TE_02_. Here, we choose *w*_2_ = 300 nm and *w*_1_ = 405 nm as the port widths so that the phases of TE_01_ and HP_02_ modes are matched.

**Figure 3:**
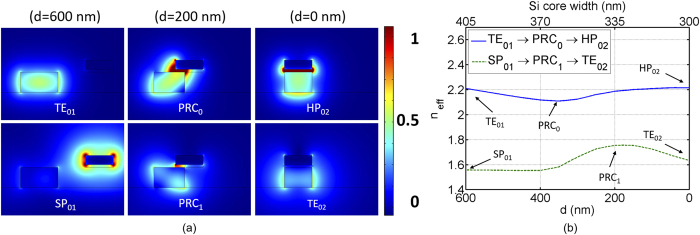
(**a**) Normalized mode profiles (|**E**|) at the cross-sections of the PRC structure when the separation distance is changed from *d* = 600 nm (port 1) to *d* = 0 nm (port 2). (**b**) Effective refractive indices as a function of separation distance *d*, and corresponding Si core width along the device. The main modes (TE_01_, PRC_0_, and HP_02_) and the secondary modes (SP_01_, PRC_1_, and TE_02_) are plotted in blue and dashed green, respectively.

Figure 3(a) shows, from left to right, the normalized mode profiles (|**E**|) at the cross-sections of the device with the separation distance *d* = 600 nm, 200 nm, and 0 nm, respectively, and the corresponding Si core width *w*_1_ = 405 nm, 335 nm, and 300 nm. Note that the case of *d* = 0 nm is the same as the cross-section of port 2, and *d* = 600 nm is the cross-section at the beginning of the conversion region. TE_01_ and SP_01_ are the TE and surface plasmon modes at the interface between port 1 and PRC, respectively, and HP_02_ and TE_02_ are the HP and TE modes at port 2, respectively. PRC_*i*_ is the *i*-th PRC mode in a hybrid state. Upper figures (TE_01_, PRC_0_, and HP_02_) are the main modes that play important roles in mode-evolution. This is an advantage over mode-coupling-based PRCs, where both PRC_0_ and PRC_1_ modes are excited for the rotation of polarization^33^,^35^.

To see the phase variations of mode-evolution in more detail, the effective refractive indices of main (blue line) and secondary (dashed green line) modes are plotted in Fig. 3(b) as a function of separation distance *d*, and corresponding Si core width along the device. Notice that TE_01_ mode is adiabatically evolved into HP_02_ mode through PRC_0_ mode (blue line), while simultaneously maintaining a large phase gap to their secondary modes (dashed green line).

## Results and Discussion

The performance of mode-evolution-based PRC with linear Si taper is evaluated by calculating the coupling factor to the HP mode (*CF*_HP_), which is defined as 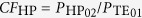, where 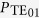 is the input power of TE_01_ mode at port 1 and 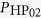 is the output power of HP_02_ mode at port 2. 3D FEM simulations are conducted by exciting TE_01_ mode at port 1; then output fields of HP_02_ mode is decomposed to calculate the 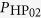
33,37.

**Figure 4:**
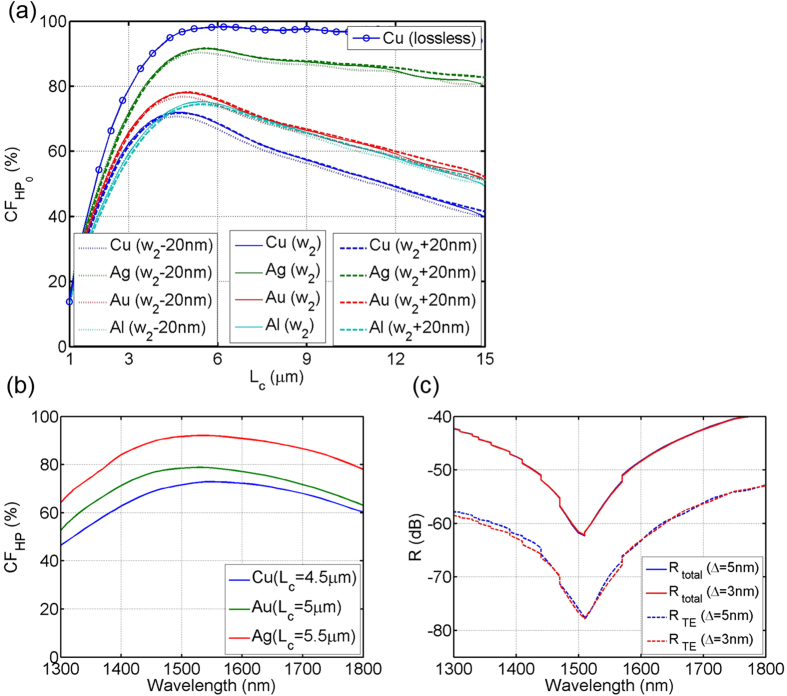
(**a**) Calculated *CF*_HP_ of the linearly tapered PRC as a function of *L*_*c*_ with different metal caps: Cu (blue), Au (green), Ag (red), and Al (cyan). Circled blue line is the case of Cu without metallic loss. Simulation results with different port widths of *w*_2_ + 20 nm and *w*_2_ − 20 nm are also presented with dashed and dotted lines, respectively. (**b**) *CF*_HP_ of the same devices as a function of *λ*_0_: Cu with *L*_*c*_ = 4.5 *μ*m (blue), Au with *L*_*c*_ = 5.0 *μ*m (green), and Ag with *L*_*c*_ = 5.5 *μ*m (red). (**c**) Calculated back-reflection power *R* spectrum of the linearly tapered PRC with different mesh grid size: Δ = 5 nm (blue lines) and Δ = 3 nm (red lines). Solid lines are the total back-reflection power (*R*_*total*_) and dashed lines are the TE component of the total back-reflection (*R*_*TE*_). Cu is used as the metal cap, and the *L*_*c*_ = 4.5 *μ*m.

Figure 4(a) shows the calculated *CF*_HP_ of the linearly tapered PRC as a function of *L*_*c*_, with different metal caps: Cu (blue), Au (green), Ag (red), and Al (cyan). Simulation results with the output port width *w*_2_ = 300 nm are plotted with plain lines, and those with different port widths *w*_2_ − 20 nm and *w*_2_ + 20 nm are also presented with dotted and dashed lines, respectively. To show the designed PRCs are truly based on mode-evolution, Cu metal cap without metallic absorption loss is also evaluated (circled blue line), and its *CF*_HP_ converges to about 97% after *L*_*c*_ = 5 *μ*m. This is distinctively different from mode-coupling-based polarization rotation, which relies on the mode interference and the coupling factors show a sinusoidal oscillation as the conversion length increases^13^,^14^,^33^,^35^. In reality when metal absorption cannot be ignored, *CF*_HP_, decreases monotonously as the *L*_*c*_ increases, after reaching a maximum point. The trend for *CF*_HP_ is similar with other metal caps, i.e., 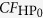 hits the maximum point then decreases as *L*_*c*_ increases; however, each metal cap shows different maximum point and descent rate. Especially, with a Ag cap, the maximum *CF*_HP_ is ~92% and the descent rate is very slow, having over 80% of *CF*_HP_ throughout the conversion lengths of 4 *μ*m to 15 *μ*m. Furthermore, simulations for *w*_2_ varied by ±20 nm show almost identical results for each material, suggesting that the devices are tolerant to fabrication variations. This is an another signature of the mode-evolution-based device and advantage over the mode-coupling-based one^30^,^31^. Also, the conversion lengths for the maximum *CF*_HP_ are ultrashort with *L*_*c*_ ~ 5 *μ*m. Typically, a mode-evolution-based device requires a long device length, e.g., hundreds of micrometers, to smoothly convert the mode adiabatically^30^,^31^; however, an ultrashort conversion length is achieved here, with a higher degree of field confinement in the HP mode

In addition to high coupling efficiency, couplers need to have broad bandwidth to cover a wide range of operating wavelengths. The spectral responses of the linearly tapered PRC are shown in Fig. 4(b), for Cu (blue), Au (green), and Ag (red) metal caps. For each metal cap, different conversion lengths are chosen, which correspond to their maximum *CF*_HP_ in Fig. 4(a): 4.5 *μ*m, 5.0 *μ*m, and 5.5 *μ*m for Cu, Au, and Ag, respectively. Notice that, in every case, the spectral ranges with *CF*_HP_ ≥ 50% are at least over 500 nm, covering the entire telecommunication bands.

In optical telecommunication systems, low back-reflection, or high return loss is also an important factor, especially for the emerging coherent transmission schemes. Despite recent advances^38^,^39^, on-chip optical isolators are not readily available, so a close-to-zero reflection is highly important if a device is to be considered for practical use. Figure 4(c) shows the calculated back reflection power of the linearly tapered PRC as a function of free space wavelengths. Cu is used as the metal cap, and the device length is set to *L*_*c*_ = 4.5 *μ*m. Solid lines are the total back-propagation power (*R*_total_), accounting all possible reflections and scatterings. Dashed lines are the TE mode component of the total back-propagation (*R*_TE_). Simulations with two different mesh grid sizes (Δ) were included: Δ = 5 nm (blue lines) and Δ = 3 nm (red lines), and the results are almost identical, suggesting high likelihood of truthfulness. Overall the total back-propagating power is below −40 dB for the entire telecommunication wavelength range of 1300–1800 nm. Note that previous HP mode couplers showed about 2 ~ 4% of back-reflections^29^, but our mode-evolution based HP coupler shows an extremely low back-reflection, because our coupling mechanism is based on the mode-evolution, which allows an adiabatic and smooth mode transition.

### Taper shape optimization

**Figure 5:**
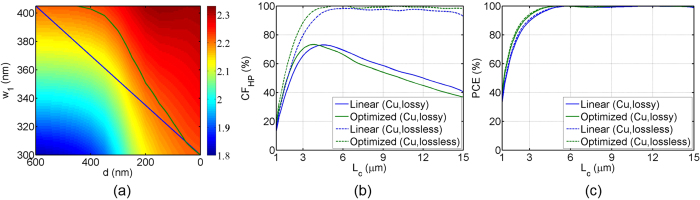
(**a**) Effective refractive index map of the fundamental modes (TE_01_, PRC_0_, or HP_02_), as functions of separation distance *d* and Si core width *w*_1_. Blue and green lines are the cases of linear and optimized (perfectly-phase-matched) taperings, respectively. Calculated (**b**) *CF*_HP_ and (**c**) *PCE* as a function of *L*_*c*_ for linear (blue) and optimized (green) taperings. Thick and dashed lines are the lossy and lossless cases, respectively.

To further optimize the mode-evolution, the effective refractive indices of the main modes are calculated as functions of both *w*_1_ and *d*, while the other geometric parameters are fixed; then the index map is plotted in Fig. 5(a), again with Cu as the metal cap. The blue line on the figure is the case of linear tapering as in Fig. 3(b), and the green curved line delineate the contour of the Si waveguide taper whose main mode (PRC_0_) has the same effective refractive index as the HP_02_ mode. Note that the lower-right corner (*d* = 0 nm and *w*_1_ = 300 nm) is the point of pure HP_02_ mode, and the end-points on top (*w*_1_ = 405 nm) are the points of pure TE_01_ mode. Keeping the separation distance *d* linearly, the Si waveguide core width *w*_1_ is tapered along the contour green line in Fig. 5(a); this tapering results in a perfect-phase-matching through the device. While there is a small phase deviation on PRC_0_ mode with a linear tapering (as the blue line in Fig. 3(b)), such deviation is removed with the contoured taper, which gives a perfect-phase-matching throughout the device. Figure 5(b) shows the calculated *CF*_HP_ as a function of *L*_*c*_ for both tapers: blue lines for linear taper and green lines for perfectly-phase-matched (optimized) taper. Cases without considering the metal absorption are also plotted with dashed lines. Compared to linearly tapered design, perfectly-phase-matched tapering has shorter *L*_*c*_ for the maximum *CF*_HP_ point, and a little bit higher coupling factor for the case without metal absorption. The shorter *L*_*c*_ of perfectly-phase-matched tapering is due to the optimized separation distance *d*, making the effective interaction length longer. In terms of the coupling factor, even in the case without metal absorption, there is about 3% of loss with linear tapering (dashed blue line); this loss is due to the scattering that comes from the phase-mismatch, and is removed by an optimized taper which gives the perfect phase-matching (dashed green line). After including absorption losses (thick lines), however, there is no appreciable improvement in coupling factor. This indicates that, in this mode-evolution-based PRC, the phase-mismatch is not a significant factor that determines the maximum coupling factor. Rather, as we’ve seen in Fig. 4(a,b), the metallic losses are the dominant factor that determines the maximum *CF*_HP_. The polarization conversion efficiency (*PCE*), which is defined as 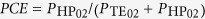, is also plotted in Fig. 5(c). Notice that the *PCE*s are almost 100% after *L*_*c*_ = 5 *μ*m for every case. This shows that there are no remaining TE mode for these mode-evolution based PRCs after a short conversion length of merely *L*_*c*_ = 5 *μ*m.

### Outline of device fabrication

In this section we describe briefly a potential route of fabrication for our proposed device. The fabrication can start from the deposition of a thin (e.g. 70 nm-thick) sacrificial silicon nitride (SiN) layer on top of the 220 nm-thick Si layer of a silicon-on-insulator (SOI) wafer. After the definition of Si waveguide via lithography and etching through the SiN and Si layers, a thick SiO_2_ layer will be deposited to cover the Si waveguides. Chemical mechanical polishing (CMP) can be applied to planarize the SiO_2_ top surface and the polishing will stop at the height of the top surface of the Si waveguide, using silicon nitride as the etch/polish stop. After removing the remaining SiN sacrificial layer, a 30 nm-thick layer of SiO_2_ can be deposited on the planarized surface with high precision in thickness to form the spacer between the Si and metal layers. The metal layers can be subsequently formed with lithography and lift-off to complete the fabrication of our device. To make the structure more robust, we propose to over clad the metal structures with SiO2 so that the metal is encapsulated between two SiO2 layers. The fabrication process does not require high-resolution lithography as all lateral features are above 300 nm, which can be achieved with any deep-UV projection lithography tools currently available in most CMOS manufacturing facilities.

## Conclusion

In summary, we present a mode-evolution-based polarization rotation and coupling scheme that has a high coupling efficiency (the maximum *CF*_HP_ is about 92% using Ag), an ultra-broad bandwidth (the spectral range with *CF*_HP_ > 64% is over 500 nm), and near-zero reflection. Also, the device length is very short (*L*_*c*_ ~ 5 *μ*m) and is tolerant to fabrication errors. The material property of plasmonic metal cap is important to the device performances such as the maximum coupling factor and the bandwidth. The Si waveguide core widths are also optimized with a perfect-phase-matching, reducing the conversion length and removing the scattering loss. This mode-evolution-based PRC scheme could meet the stringent requirements in optical communications systems and should be useful in HP-waveguide-based applications such as electro-optical modulators^15^, nano-lasers^10^, ring-resonators^19^, nonlinear optical devices^20^, and quantum plasmonic devices^40^,^41^,^42^.

## Additional Information

**How to cite this article**: Kim, S. and Qi, M. Mode-evolution-based polarization rotation and coupling between silicon and hybrid plasmonic waveguides. *Sci. Rep.* **5**, 18378; doi: 10.1038/srep18378 (2015).

## References

[b1] Lipson M. Guiding, modulating, and emitting light on silicon-challenges and opportunities. J. Lightwave Technol. 23, 4222 (2005).

[b2] Kirchain R. & Kimerling L. A roadmap for nanophotonics. Nature Photon. 1, 303–305 (2007).

[b3] Soref R. The past, present, and future of silicon photonics. IEEE J. Sel. Topics Quantum Electron 12, 1678–1687 (2006).

[b4] Jalali B. & Fathpour S. Silicon photonics. J. Lightwave Technol. 24, 4600–4615 (2006).

[b5] Vlasov Y. A., O’Boyle M., Hamann H. F. & McNab S. J. Active control of slow light on a chip with photonic crystal waveguides. Nature 438, 65–69 (2005). 16267549 10.1038/nature04210

[b6] Sorger V. J., Oulton R. F., Ma R.-M. & Zhang X. Toward integrated plasmonic circuits. MRS bulletin 37, 728–738 (2012).

[b7] Brongersma M. L. & Shalaev V. M. Applied physics the case for plasmonics. Science 328, 440–441 (2010). 20413483 10.1126/science.1186905

[b8] Alam M. Z., Aitchison J. S. & Mojahedi M. A marriage of convenience: Hybridization of surface plasmon and dielectric waveguide modes. Laser Photon. Rev. 8, 394–408 (2014).

[b9] Oulton R. F., Sorger V. J., Genov D., Pile D. & Zhang X. A hybrid plasmonic waveguide for subwavelength confinement and long-range propagation. Nature Photon. 2, 496–500 (2008).

[b10] Oulton R. F. *et al.* Plasmon lasers at deep subwavelength scale. Nature 461, 629–632 (2009). 19718019 10.1038/nature08364

[b11] Guan X., Wu H., Shi Y., Wosinski L. & Dai D. Ultracompact and broadband polarization beam splitter utilizing the evanescent coupling between a hybrid plasmonic waveguide and a silicon nanowire. Opt. Lett. 38, 3005–3008 (2013). 24104633 10.1364/OL.38.003005

[b12] Kim S. & Qi M. Copper nanorod array assisted silicon waveguide polarization beam splitter. Opt. Express 22, 9508–9516 (2014). 24787839 10.1364/OE.22.009508 PMC4083047

[b13] Caspers J. N., Alam M. & Mojahedi M. Compact hybrid plasmonic polarization rotator. Opt. Lett. 37, 4615–4617 (2012). 23164856 10.1364/ol.37.004615

[b14] Caspers J. N., Aitchison J. S. & Mojahedi M. Experimental demonstration of an integrated hybrid plasmonic polarization rotator. Opt. Lett. 38, 4054–4057 (2013). 24321921 10.1364/OL.38.004054

[b15] Sorger V. J., Lanzillotti-Kimura N. D., Ma R.-M. & Zhang X. Ultra-compact silicon nanophotonic modulator with broadband response. Nanophotonics 1, 17–22 (2012).

[b16] Boltasseva A. & Atwater H. A. Low-loss plasmonic metamaterials. Science 331, 290–291 (2011). 21252335 10.1126/science.1198258

[b17] West P. R. *et al.* Searching for better plasmonic materials. Laser Photon. Rev. 4, 795–808 (2010).

[b18] Dai D. & He S. Low-loss hybrid plasmonic waveguide with double low-index nano-slots. Opt. Express 18, 17958–17966 (2010). 20721182 10.1364/OE.18.017958

[b19] Ketzaki D. A., Tsilipakos O., Yioultsis T. V. & Kriezis E. E. Electromagnetically induced transparency with hybrid silicon-plasmonic traveling-wave resonators. J. Appl. Phys. 114, 113107 (2013).

[b20] Pitilakis A. & Kriezis E. E. Highly nonlinear hybrid silicon-plasmonic waveguides: analysis and optimization. J. Opt. Soc. Am. B 30, 1954–1965 (2013).

[b21] Mu J. *et al.* Hybrid nano ridge plasmonic polaritons waveguides. Appl. Phys. Lett. 103, 131107 (2013).

[b22] Liang H., Soref R., Mu J., Li X. & Huang W.-P. Long range mid-infrared propagation in si and ge hybrid plasmonic-photonic nano-ribbon waveguides. Opt. Express 22, 28489–28499 (2014). 25402091 10.1364/OE.22.028489

[b23] Mu J., Soref R., Kimerling L. C. & Michel J. Silicon-on-nitride structures for mid-infrared gap-plasmon waveguiding. Appl. Phys. Lett. 104, 031115 (2014).

[b24] Soref R. Mid-infrared photonics in silicon and germanium. Nature Photon. 4, 495–497 (2010).

[b25] Hu J., Meyer J., Richardson K. & Shah L. Feature issue introduction: mid-ir photonic materials. Opt. Mater. Express 3, 1571–1575 (2013).

[b26] Wang J. *et al.* Monolithically integrated, resonant-cavity-enhanced dual-band mid-infrared photodetector on silicon. Appl. Phys. Lett. 100, 211106 (2012).

[b27] Briggs R. M., Grandidier J., Burgos S. P., Feigenbaum E. & Atwater H. A. Efficient coupling between dielectric-loaded plasmonic and silicon photonic waveguides. Nano Lett. 10, 4851–4857 (2010). 21028908 10.1021/nl1024529

[b28] Emboras A. *et al.* Efficient coupler between silicon photonic and metal-insulator-silicon-metal plasmonic waveguides. Appl. Phys. Lett. 101, 251117–251117 (2012).

[b29] Song Y., Wang J., Li Q., Yan M. & Qiu M. Broadband coupler between silicon waveguide and hybrid plasmonic waveguide. Opt. Express 18, 13173–13179 (2010). 20588445 10.1364/OE.18.013173

[b30] Watts M. & Haus H. Integrated mode-evolution-based polarization rotators. Opt. Lett. 30, 138–140 (2005). 15675692 10.1364/ol.30.000138

[b31] Watts M., Haus H. & Ippen E. Integrated mode-evolution-based polarization splitter. Opt. Lett. 30, 967–969 (2005). 15906972 10.1364/ol.30.000967

[b32] Watts M. R. *et al.* Towards integrated polarization diversity: design, fabrication and characterization of integrated polarization splitters and rotators. In *Optical Fiber Communication Conference*, PDP11 (Optical Society of America, 2005).

[b33] Kim S. & Qi M. Polarization rotation and coupling between silicon waveguide and hybrid plasmonic waveguide. Opt. Express 23, 9968–9978 (2015). 25969038 10.1364/OE.23.009968 PMC4523377

[b34] Luo Y. *et al.* On-chip hybrid photonic-plasmonic light concentrator for nanofocusing in an integrated silicon photonics platform. Nano Lett. 15, 849–856 (2015). 25562706 10.1021/nl503409k

[b35] Jin L., Chen Q. & Wen L. Mode-coupling polarization rotator based on plasmonic waveguide. Opt. Lett. 39, 2798–2801 (2014). 24784106 10.1364/OL.39.002798

[b36] Johnson P. B. & Christy R.-W. Optical constants of the noble metals. Phys. Rev. B 6, 4370 (1972).

[b37] Yariv A. & Yeh P. Photonics: Optical Electronics in Modern Communications (Oxford University Press, Inc., 2006).

[b38] Fan L. *et al.* An all-silicon passive optical diode. Science 335, 447–450 (2012). 22194410 10.1126/science.1214383 PMC5563475

[b39] Bi L. *et al.* On-chip optical isolation in monolithically integrated non-reciprocal optical resonators. Nature Photon. 5, 758–762 (2011).

[b40] de Leon N. P., Lukin M. D. & Park H. Quantum plasmonic circuits. IEEE J. Sel. Topics Quantum Electron. 18, 1781–1791 (2012).

[b41] Tame M. *et al.* Quantum plasmonics. Nature Phys. 9, 329–340 (2013).

[b42] Fakonas J. S., Lee H., Kelaita Y. A. & Atwater H. A. Two-plasmon quantum interference. Nature Photon. 8, 317–320 (2014).

